# Invalid Children's and Association

**Published:** 1904-10-22

**Authors:** 


### INVALID CHILDREN'S AID ASSOCIATION.

We mentioned, at the time when it was held at the Guildhall during the summer, an important conference which was organised by the London Invalid Children's Aid Association, and have now to call attention to a modest but extremely valuable *compte rendu*, in the shape of an unpretending pamphlet of 60 pages, which has just been issued by the association, and which contains the most important of the papers read at the conference. We endeavoured at the time to do justice to Dr. Newsholme's paper on the social aspects of the prevention of illness, and will therefore pass on to one contributed by Dr. Cautley on cow's milk as a food in infancy, which is worthy of being studied and preserved in every household of which children form a part. It places in their true light the claims of a good deal of the so-called "sterilisation" and "humanisation" to which cow's milk is subjected; and shows how little either the professions of milk sellers for profit, or the well-intended, but often ignorant, efforts of municipalities are to be relied upon for the preservation of infantile health and digestion. The prevention of tuberculosis among children is dealt with by Dr. Raw, the results obtained by the Association in cases of joint disease are described by Mr. Howell Evans, and the importance of removing children suffering from surgical tuberculosis from the atmosphere and surroundings of large towns is dwelt upon by Mr. Tubby. The Duchess of Sutherland contributes an extremely interesting article, with the one fault of being too short, upon the work of the Cripples Guild in the Potteries; and other subjects treated are the apprenticeship and working capacity of cripples, and the educational handling of physically and mentally defective children and of epileptics. The importance of the schools for cripples may be estimated from the fact that, although but a short time has elapsed since such schools were established by Mrs. Humphry Ward, and taken over by the educational authorities, they now provide for the teaching of 900 children in London alone. We can cordially recommend the modest report of which we are speaking, not only as a record of good work, but also as a mine of condensed and accurate information on the subjects to which it relates. It is to be obtained at the office of the Association, 8 Henrietta Street, Covent Garden.

